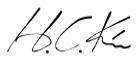# Happy New Year 2022 from *Epidemiology and Health* (*epi*H)

**DOI:** 10.4178/epih.e2022001

**Published:** 2021-12-28

**Authors:** Hyeon Chang Kim

**Affiliations:** Department of Preventive Medicine, Yonsei University College of Medicine, Seoul, Korea

On behalf of the editorial board members of *Epidemiology and Health* (*epi*H), I would like to express my gratitude to the authors and readers for their contributions last year. In fact, 2021 was among the most memorable years in the history of *epi*H. Last July, *epi*H was included in Clarivate’s Journal Citation Report for the first time. This occurred not long after I was appointed editor-in-chief on June 1. *epi*H received a good Journal Impact Factor (JIF) of 3.282, ranking 142nd out of 374 journals in the “Public, Environmental & Occupational Health” category. *epi*H was founded in 1979 under the name of *Korean Journal of Epidemiology*, and 30 years later, it was reborn as an English-language journal by changing its name to *Epidemiology and Health* (*epi*H). I would like to express my deepest gratitude to the 7 former editors-in-chief for their superb leadership. In particular, Moran Ki, who served as editor-in-chief from June 2013 to May 2021, made remarkable achievements by having *epi*H listed in the most important journal directories, including MEDLINE, Scopus, Directory of Open Access Journals (DOAJ), Korea Citation Index (KCI), Emerging Sources Citation Index (eSCI), and Science Citation Index Expanded (SCIE).

In addition to receiving its first JIF, other remarkable developments took place in 2021. *epi*H has become an important international journal in the fields of epidemiology and public health. In 2021, a total of 784 manuscripts were received from 76 countries, of which 645 (82.3%) were submitted from outside of Korea. Among them, 103 articles, reflecting a 13.1% acceptance rate, were published, including 62 from Korea, 18 from other Asian countries, 9 from America, 8 from Europe, and 6 from Africa. By publishing 23 research papers and perspectives on COVID-19 issues, *epi*H has contributed to coping with the public health crisis caused by the pandemic.

In addition to acknowledging the contributions made by the authors who have submitted their work to *epi*H, I would also like to recognize with gratitude the dedication of reviewers and editors. Last year, 187 scholars served as external reviewers for submitted papers. In particular, I would like to express my great gratitude to professors Daejung Kim (who reviewed 7 papers), Sanghyuk Bae (who reviewed 7 papers), and Boyoung Park (who reviewed 6 papers). I would also like to express sincere thanks to the 24 editors who were in charge of the internal and external review process of the submitted papers. Associate editor Jong-Hun Kim managed as many as 96 papers; Hyeon Chang Kim, Hyesook Park, and Hae-Kwan Cheong managed more than 80 papers; and Moran Ki and Hyeon Woo Yim managed more than 50 papers. I would also like to thank the editorial staff members, Young-Ju Lee and Young Ju Choi, for handling the rapidly increasing number of papers without any problems. Furthermore, I deeply appreciate Soon Young Lee, president of the Korean Society of Epidemiology, and the board of directors for their full support.

Hopefully, in 2022, we can successfully overcome the COVID-19 pandemic, restore the collapsing healthcare system, and reverse worsening health inequalities. Finally, I would like to express my sincere respect to my fellow epidemiologists and health care professionals around the world who are working hard to achieve these goals.